# Impact of androgen deprivation therapy on apparent diffusion coefficient and T2w MRI for histogram and texture analysis with respect to focal radiotherapy of prostate cancer

**DOI:** 10.1007/s00066-018-1402-3

**Published:** 2018-11-26

**Authors:** M. Daniel, P. Kuess, P. Andrzejewski, T. Nyholm, T. Helbich, S. Polanec, F. Dragschitz, G. Goldner, D. Georg, P. Baltzer

**Affiliations:** 1 Christian Doppler Laboratory for Medical Radiation Research for Radiation Oncology, Medical University of Vienna, Vienna, Austria; 2 Department of Radiotherapy, Comprehensive Cancer Center, Medical University of Vienna/Vienna General Hospital, Vienna, Austria; 3 Medical Physics, EBG MedAustron GmbH, Wiener Neustadt, Austria; 4 Department of Radiation Sciences, Umeå University, Umeå, Sweden; 5 Department of Biomedical Imaging and Image-Guided Therapy, Medical University of Vienna/Vienna General Hospital, Vienna, Austria

**Keywords:** Radiomics, Tissue characterization, Boost volume, Quantitative imaging, ADC, Radiomics, Gewebecharakterisierung, Boost-Volumen, Quantitative Bildgebung, ADC

## Abstract

**Purpose:**

Accurate prostate cancer (PCa) detection is essential for planning focal external beam radiotherapy (EBRT). While biparametric MRI (bpMRI) including T2-weighted (T2w) and diffusion-weighted images (DWI) is an accurate tool to localize PCa, its value is less clear in the case of additional androgen deprivation therapy (ADT). The aim of this study was to investigate the value of a textural feature (TF) approach on bpMRI analysis in prostate cancer patients with and without neoadjuvant ADT with respect to future dose-painting applications.

**Methods:**

28 PCa patients (54–80 years) with (*n* = 14) and without (*n* = 14) ADT who underwent bpMRI with T2w and DWI were analyzed retrospectively. Lesions, central gland (CG), and peripheral zone (PZ) were delineated by an experienced urogenital radiologist based on localized pre-therapeutic histopathology. Histogram parameters and 20 Haralick TF were calculated. Regional differences (i. e., tumor vs. PZ, tumor vs. CG) were analyzed for all imaging parameters. Receiver-operating characteristic (ROC) analysis was performed to measure diagnostic performance to distinguish PCa from benign prostate tissue and to identify the features with best discriminative power in both patient groups.

**Results:**

The obtained sensitivities were equivalent or superior when utilizing the TF in the no-ADT group, while specificity was higher for the histogram parameters. However, in the ADT group, TF outperformed the conventional histogram parameters in both specificity and sensitivity. Rule-in and rule-out criteria for ADT patients could exclusively be defined with the aid of TF.

**Conclusions:**

The TF approach has the potential for quantitative image-assisted boost volume delineation in PCa patients even if they are undergoing neoadjuvant ADT.

**Electronic supplementary material:**

The online version of this article (10.1007/s00066-018-1402-3) contains supplementary material, which is available to authorized users.

## Zusammenfassung

**Zielsetzung:**

Eine exakte Lokalisation des Prostatakarzinoms (PCa) ist für die Bestrahlungsplanung von externer Photonentherapie mit integriertem Boost essenziell. Während die biparametrische Magnetresonanztomographie (bpMRT) mit T2-gewichteter (T2w) und Diffusionsbildgebung (DWI) als genaue Methode zur PCa-Lokalisierung gilt, ist deren Bedeutung bei zusätzlicher Hormontherapie („androgen deprivation therapy“, ADT) unklar. Ziel der Studie ist die Evaluierung des Nutzens eines texturanalysebasierten Ansatzes bei bpMRT von Prostatakarzinompatienten mit und ohne ADT im Hinblick auf zukünftige Dose-Painting-Anwendungen.

**Methoden:**

Die bpMRT-Daten (T2w und DWI) von 28 Prostatakarzinompatienten (54–80 Jahre; 14 mit ADT) wurden retrospektiv analysiert. Läsionen, zentrales Drüsengewebe (CG) und periphere Zone (PZ) wurden von einem erfahrenen Radiologen unter Berücksichtigung der lokalisierten prätherapeutischen Histopathologie deliniert. Histogrammparameter und 20 Haralick-TF wurden berechnet. Für alle Bildgebungsparameter wurden die regionalen Unterschiede (z. B. Läsion vs. PZ, Läsion vs. CG) analysiert. Mit der ROC(„Receiver-operating characteristic“)-Analyse wurde die Leistung hinsichtlich Differenzierbarkeit von PCa und gesundem Gewebe gemessen und Parameter mit der besten Unterscheidungskraft in beiden Patientengruppen identifiziert.

**Ergebnisse:**

In der Gruppe ohne ADT waren die resultierenden Sensitivitäten äquivalent oder höher für die TF, während die Histogrammparameter eine höhere Spezifität aufwiesen. In der ADT-Gruppe übertraf die Genauigkeit der TF jedoch die der herkömmlichen Histogrammparameter sowohl hinsichtlich Sensitivität als auch Spezifität. Einschluss- und Ausschlusskriterien konnten für die ADT-Patienten nur mit Hilfe von TF definiert werden.

**Schlussfolgerung:**

Der TF-Ansatz hat das Potenzial zur quantitativen Definition des Boost-Volumens in PCa-Patienten, unabhängig von einer neoadjuvanten ADT.

## Introduction

Defining boost volumes for prostate cancer (PCa) patients is a major step towards personalized radiation oncology with the overall goal of increasing tumor control probability. Recently, the FLAME trial [1, 2] focused on the evaluation of 5‑year freedom from biochemical failure as well as toxicity when applying a boosting method. Also, several other studies investigated the feasibility of boosting the dominant intraprostatic lesion with various methods in patients with advanced prostate cancer, which indicated superior local control [3–7]. The presented results suggest that the implementation of this approach in intermediate and high-risk prostate cancer patients improves tumor control while keeping side effects at a reasonable level. Although some groups based the delineation of the boost volume on PET imaging [4, 5], multiparametric (mp) magnetic resonance imaging (MRI) has become the preferred method for this purpose [8, 9]. Recently, simplified biparametric (bp) protocols which do not require contrast medium injection have been demonstrated to yield similar results to full protocols, thus allowing shorter acquisition times and a broader access to this imaging modality [10]. Moreover, for the external beam radiotherapy (EBRT) planning in prostate cancer it is recommended to base prostate gland delineation on MRI [11, 12].

Numerous independent studies found that prescribing androgen deprivation therapy (ADT) in addition to EBRT is associated with improved survival for patients with intermediate-risk and high-risk disease [13]. In today’s clinical practice, the combination of ADT and radiotherapy is considered as gold-standard treatment [14, 15]. This has, however, a drawback with regard to imaging, since ADT leads to an increase in tumor apparent diffusion coefficient (ADC) and thus a decreased prostate cancer conspicuity [16–19]. Data on the influence of ADT on tumor delineation is sparse and it remains uncertain whether boost volumes for upcoming focal irradiation techniques can be defined for patients who had neoadjuvant ADT prior to radiotherapy by the same methods as those that are currently employed for patients without ADT.

With the rise of radiomics [20], many studies investigated the use of textural features (TF) for prostate cancer patients (e. g., risk categorization on active surveillance [21], predictive models of biochemical recurrence after therapy [22, 23], combining histogram-based and textural parameters for a better differentiation between tumor and healthy tissue [24]). Furthermore, the use of computer-aided or machine learning approaches is strongly increasing in the context of tumor auto-delineation [25–27]. The value of radiomics for prostate cancer detection and delineation in ADT patients has not yet been addressed by empirical research.

The aim of this study was to investigate the additive value of a TF approach compared to histogram-based parameters on bpMRI analysis by means of separating tumor from healthy tissue for prostate cancer patients with or without ADT.

## Materials and methods

### Patient data and imaging

This retrospective and institutional review board-approved study was performed on a subgroup of patients receiving prostate radiotherapy at the Department of Radiotherapy, Medical University of Vienna/Vienna General Hospital between 11/2013 and 01/2017. More specifically, out of in total 360 primary PCa RT patients, 28 patients who fulfilled the following criteria were included: biopsy-proven primary prostate cancer, (neo-)adjuvant ADT prescribed (approximately 50% of the PCa patients), and bpMRI before EBRT (15% of the patients). Patients who did not receive ADT at any timepoint at all were excluded to ensure patient group homogeneity and to reduce clinical selection bias. Patients who underwent re-irradiations or had any additional secondary indications were excluded too.

**Fig. 1:**
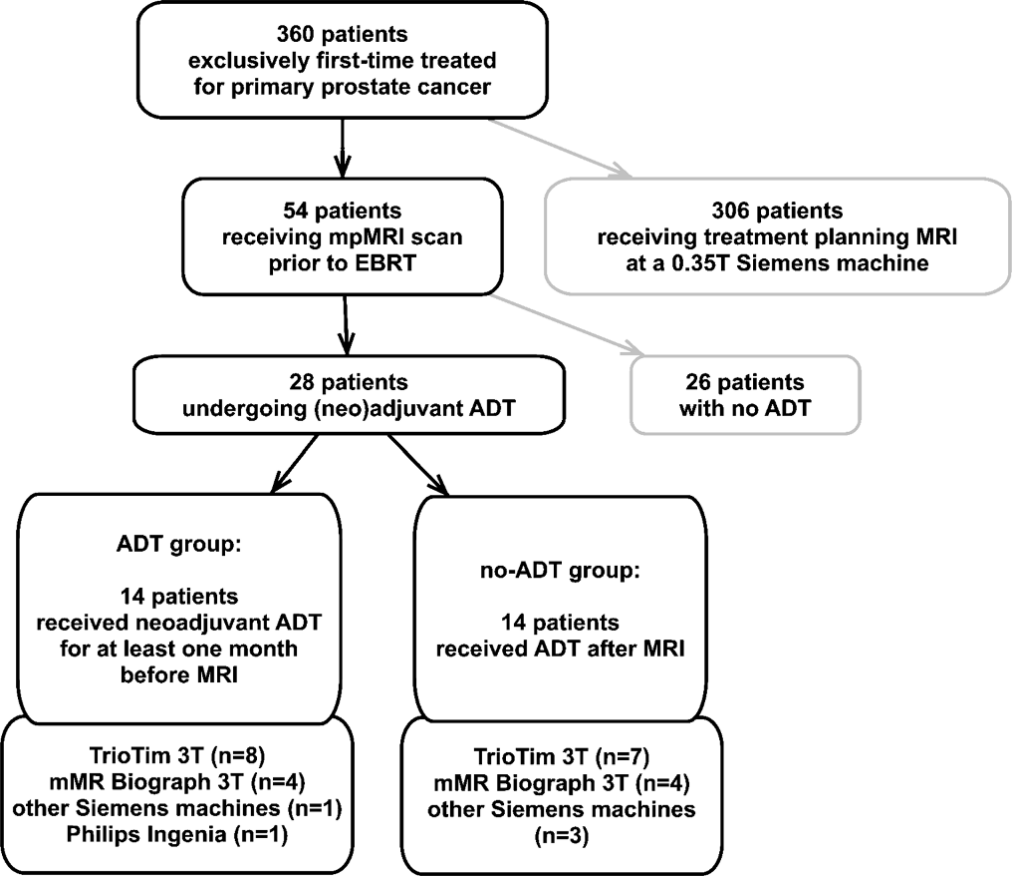
Flowchart of the data inclusion process and the assignment to patient subgroups. *Grey boxes *symbolize patients who were not further analyzed in our study. The MR scanners used for data acquisition (Siemens Healthineers, Erlangen, Germany; Philips Medical Systems, Hamburg, Germany) are listed in the charts attached to the separate subgroups

The median patient age was 73 years (range 54–80 years), the median Gleason score was 7 (range 6–9), and the median PSA value before radiotherapy was 11 ng/ml (range 5–551 ng/ml). All patients received EBRT with a prescription dose of at least 78 Gy (2 Gy single dose) or a prescription dose of 73 Gy (2.4 and 2.6 Gy single dose). Dependent on their risk staging, patients additionally received a 45 (*n* = 3) or 50.4 Gy (*n* = 23) dose to the adjacent lymph nodes. 14 patients received ADT before MR imaging (“ADT group”). The median time period between start of ADT and bpMRI was 4 months (range 1–118 months). Three patients received ADT a long time (10, 8, and 5 years, respectively) before the clinical decision was made to start radiotherapy. It has to be highlighted that all patients in this group were still under ADT at the time of imaging. The remaining 14 patients form the “no-ADT group” and started ADT only after their bpMRI scan (Fig. 1).

The MRI protocol included a transversal T2-weighted turbo spin-echo sequence (T2w) and diffusion-weighted single-shot echo planar imaging sequence, and the derived apparent diffusion coefficient map (ADC) that was automatically calculated by the scanner software using a pixelwise monoexponential analysis of the diffusion-weighted images. MRI scans were acquired at different 3T units, including a hybrid PET/MR scanner. Details on the imaging protocols can be found in the supplementary table S1.

### Image processing

#### ROI definition

The whole prostate gland, its peripheral zone (PZ), central gland (CG), and focal lesion(s) were delineated on the T2w images by an experienced radiologist (>10 years’ experience in uroradiology). The ADC information was additionally used for delineation support, which is current practice in clinical routine. Delineation was performed using the Mirada^RTx^ (Mirada Medical, Oxford, UK) system. The reader was blinded to whether a patient started ADT before the scan. Although ADT lowers tumor conspicuity [17], in all patients, a primary lesion could be identified. For the PET/MR hybrid scanner cases (*n* = 8), PET data could be consulted for tumor delineation as well (^11^C Acetat, *n* = 2; PSMA, *n* = 1; Ga-DOTA-peptide, *n* = 5). Further, the delineation was cross-validated with pre-therapeutic biopsy data. Biopsies were either template biopsies (24 specimens, *n* = 4), TRUS-guided systematic biopsies (8–10 specimens, *n* = 2; 12–14 specimens, *n* = 16), or MRI-guided in-bore biopsies (2–4 specimens, *n* = 6). Six patients showed multi-focal disease. Tumor volumes were summed up and analyzed jointly for these cases. For the analysis of PZ and CG, tumor volumes were subtracted. Image pre-processing as described below was performed utilizing the MICE Toolkit® (© 2018 NONPI Medical AB, Umeå, Sweden). Histogram equalization was performed with the MATLAB (Mathworks, Natick, Massachusetts) function *histeq* through an integrated MATLAB node in the MICE Toolkit®.

#### T2w pre-processing

All image data were corrected for the MR bias field via N4 algorithm using the BSpline upsample interpolator [28] (FWHM = 0.15, number of control points = 4, spline order = 3, bins = 200, Wiener filter noise = 0.01, shrink factor = 2). A convergence threshold of 0.002 was found to be most suitable for the present data. Images were cut to a region of interest (ROI; defined by a box enclosing the prostate plus a 1 cm margin) for each patient in order to ensure the equivalent anatomical regions for the normalization step. The image histograms were truncated to the 98% and 2% percentile (perc) and the grey values were normalized to 0 to 350 (roughly corresponding to the patient-averaged prostate maximum value). Histogram parameters (mean, 95perc, 5perc, skewness, kurtosis) were extracted from these data. For the TF analysis, histogram equalization was additionally performed in advance.

#### ADC pre-processing

A rigid registration (Elastix, ITK implemented in MICE) was performed between T2w and ADC image series to minimize errors due to distortion and/or internal organ movement caused by gas pockets in the rectum. For this purpose, the whole prostate gland plus 3 cm margin was set as the ROI. In difficult cases, the automatic registration step was manually tweaked so that the delineated focal lesion aligned well with the area of restricted diffusivity. For cases with distortions, an additional manual adaptation of the PZ was necessary. One ADC dataset had to be excluded due to artifacts caused by a hip implant. To allow inter-patient comparison, the grey values of the image data were truncated to zero and the value of the urine signal; additionally, they were normalized to 0 − 2.5 ∙ 10^−3^ mm^2^/s (corresponding to the patient-averaged bladder mean). High ADC regions inside the CG were excluded from the analysis by thresholding for patients with cysts or a catheter. Finally, histogram equalization was performed in advance to the TF analysis.

#### Analysis

**Fig. 2:**
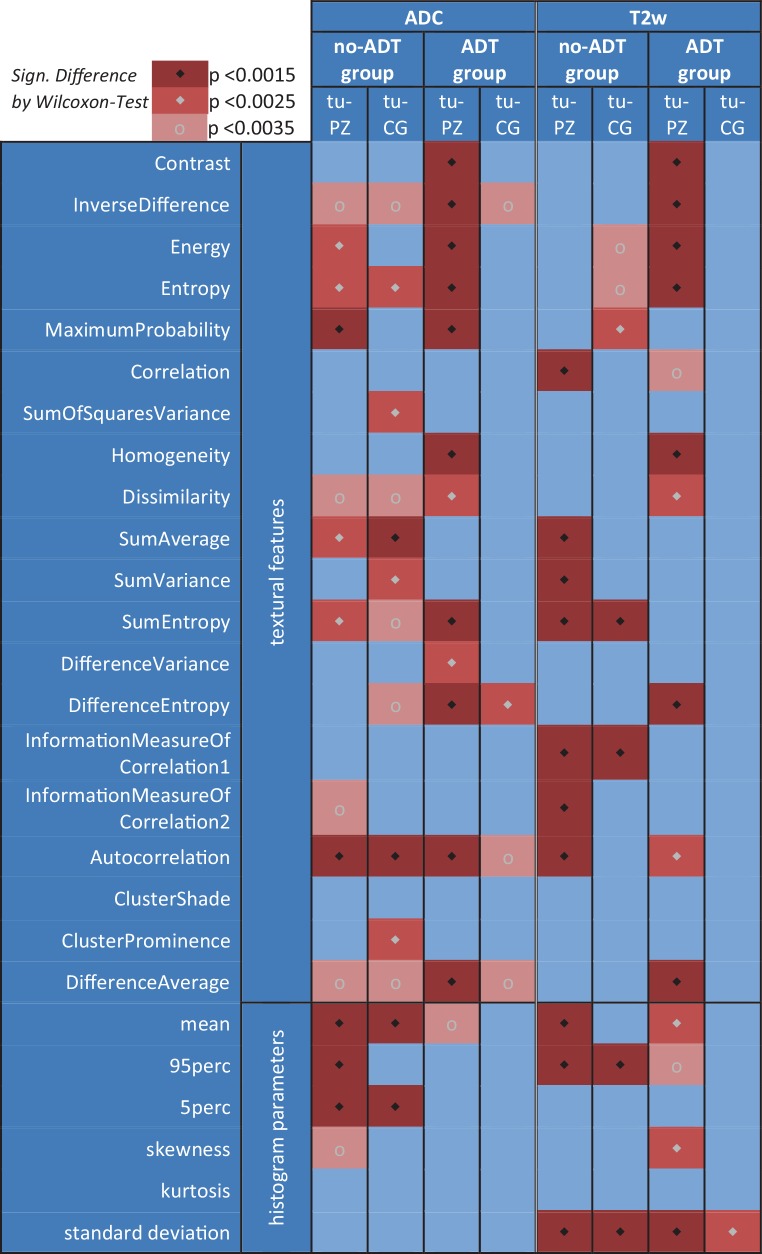
Regional comparison of all parameters (textural features and histogram parameters) with different levels of *p*-value significance. *ADC* apparent diffusion coefficient, *T2w* T2 weighted magnetic resonance tomography, *ADT* androgen deprivation therapy, *tu* tumor, *PZ* peripheral zone, *CG* central gland

The differences in clinical parameters of the two patient groups were assessed. The histogram analysis of T2w and ADC encompassed the patient-wise mean, 95perc, 5perc, standard deviation (SD), skewness, and kurtosis of the lesion, the PZ and the CG. Twenty Haralick TF (Fig. 2) of both modalities were calculated for the same regions as well using the MICE Toolkit®. The respective grey-level co-occurrence matrix (GLCM) was calculated with 16 bins in all four directions (horizontal, vertical, diagonal left, diagonal right). The grey-level invariant features [29, 30], i. e., TF that are free of the general dependence on the binning number, were then derived from the averaged GLCM. Regional differences (i. e., lesion vs. PZ, lesion vs. CG) were analyzed for all imaging parameters.

### Statistical analysis

Statistical analysis was performed in SPSS 24.0 (SPSS Inc. IBM Analytics). Inter-patient group differences were assessed with a Mann–Whitney U test for the focal lesion, the PZ, and the CG. Regional differences were analyzed via a Wilcoxon rank sum test. Bonferroni correction was applied to all tests to address the multiplicity problem. The resulting alpha value was 0.0015 (one-sided). For feature selection for the tissue classification of both subgroups, logistic regression (backward: LR) was performed. Goodness of fit statistics were calculated by the Hosmer–Lemeshow test. ROC analysis was performed on the selected features. Additionally, a decision-tree analysis (exhaustive chi-squared automated interaction detection method, CHAID) with 10-fold cross-validation was performed for the delineated regions (i. e., lesion vs. PZ, lesion vs. CG) of both subgroups to obtain rule-in and rule-out criteria. The applied parameters are as follows: a minimum number of cases of 10 for the parent node, 5 for the child node, and a Bonferroni-corrected significance level for splitting nodes of 0.05.

## Results

No significant differences were found between the ADT and no-ADT patient groups for PSA_initial_, ROI volumes, and Gleason scores (GS). PSA_nadir_ was on average 0.1 ng/ml higher for ADT patients (*p* = 0.019; supplementary table S2). When comparing the two subgroups, neither the histogram parameters nor the textural features differed significantly pairwise for any of the delineated regions. Only trends (*p* < 0.05) to lower values in the ADT group could be observed for mean, SD, and 95perc of ADC of the CG and T2w kurtosis of the focal lesion.

The T2w_mean_ overall patient mean (first quartile; third quartile) was 101 (80; 113), 153 (121; 183), and 120 (98; 136) in lesion, PZ, and CG, respectively. Overall ADC_mean_ patient mean values for these three regions were 1.01 (0.79; 1.14), 1.35 (1.26; 1.47), 1.24 (1.03; 1.34) [∙ 10^−3^ mm^2^/s].

Intra-group comparison of the imaging parameters of lesion, PZ, and CG resulted in significant differences between healthy and tumor tissue (Fig. 2). In more detail, in the no-ADT group, three histogram-based features were significantly different for lesion and PZ while for the ADT group no significant differences were observed. On the other hand, two and ten of the ADC TF showed significant differences between lesion and PZ in both the no-ADT and the ADT group, respectively. Similar findings were identified for the T2w-derived TF, i. e., for both patient groups; seven TF showed significant differences, albeit different ones. In the no-ADT group, Correlation, Sum Average, SumVariance, SumEntropy, Autocorrelation, and InformationMeasureOfCorrelation1 and 2, while in the ADT group, Contrast, InverseDifference, Energy, Entropy, Homogeneity, DifferenceEntropy, and DifferenceAverage (*p* < 0.0015; Fig. 2) were statistically significant.

**Fig. 3:**
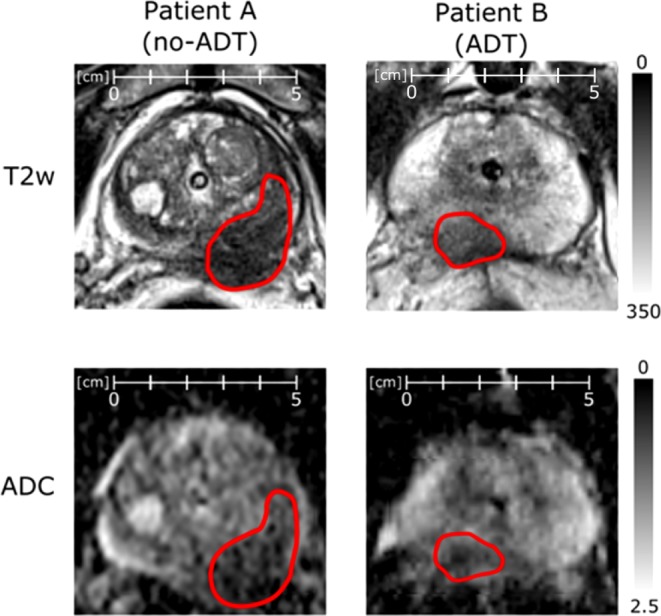
Side-by-side comparison of normalized T2w and ADC images of representative patients of each subgroup. Both patients were scanned on a Siemens mMR Biograph. Tumor conspicuity is empirically lower for patient B in both modalities compared to patient A. *ADC* apparent diffusion coefficient, *T2w* T2 weighted magnetic resonance tomography, *ADT* androgen deprivation therapy

**Fig. 4:**
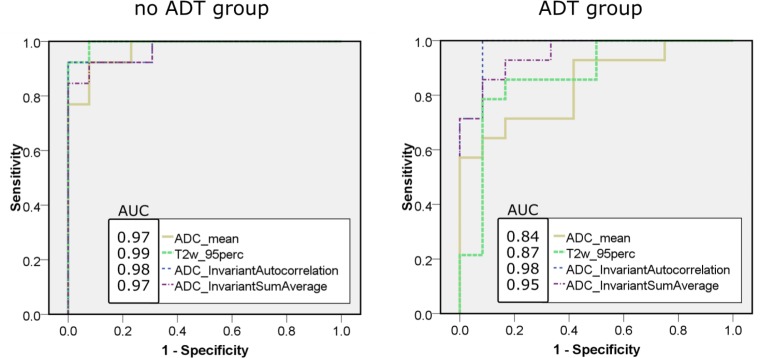
ROC curves of the top performing parameters for classifying PZ and lesion: ADC_mean, T2w_95perc (histogram parameters); ADC_Autocorrelation, ADC_Sum Average (TF). Sensitivity and specificity are lower in the ADT group compared to the no-ADT group. TF outperform histogram parameters in the ADT group. ROC curves classifying CG and lesion have a similar appearance. *ADC* apparent diffusion coefficient, *T2w* T2 weighted magnetic resonance tomography, *ADT* androgen deprivation therapy

Representative patient datasets with and without ADT are presented in Fig. 3. Patient A did not receive ADT at the time of the MRI and patient B was 2 months under ADT before the MRI. Focal lesion borders are more washed-out in patient B, indicating lower tumor conspicuity. ROC curves of the two top-performing histogram parameters and TF each are plotted in Fig. 4. For the no-ADT group, sensitivity in the PZ of T2w_95perc and TF was equivalent, while specificity was lower for the TF (T2w_95perc: 0.93, TF: 0.66). Sensitivity in the CG increased from 0.75 to 0.93 by taking textural features into account, while specificity was superior in T2w_95perc compared to TF (0.93 cf. 0.60). Overall sensitivity and specificity were lower in the ADT group. However, in the ADT group, TF outperformed the conventional histogram parameters in both specificity and sensitivity for both CG and PZ. Decision-tree classification diagrams are presented in the supplementary figure S1. The corresponding rule-in/rule-out criteria for the present patient groups derived from decision-tree classification are listed in Table 1.

**Table 1:** Rule-in and rule-out criteria from decision tree classification. When two criteria are stated, it is meant as a combined criterion. For the ADT group no rule-in criterion for PZ and no rule-out criterion for CG could be identified. The complementary two criteria are based on the TF Autocorrelation

	no-ADT				ADT			
	PZ		CG		PZ		CG	
**RULE-OUT** (100% healthy)	T2w_mean	>133	T2w_95perc	>204	ADC_InvariantAutocorrelation	>0.645	–	
	OR		OR					
	T2w_mean & ADC_mean	<133 >1.17	T2w_95perc & ADC_5perc	<204 >0.84				
**RULE-IN** (100% tumor)	T2w_mean &ADC_mean	<133 <1.17	T2w_95perc &ADC_5perc	<204 <0.84	–		ADC_InvariantAutocorrelation	<0.333

*ADC* apparent diffusion coefficient, *T2w* T2 weighted magnetic resonance tomography, *ADT* androgen deprivation therapy, *PZ* peripheral zone, *CG* central gland

## Discussion

In this study, the feasibility of prostate tissue classification into healthy and tumor tissue has been explored based on a new textural feature approach in patients with and without ADT. TF differed between tumor and surrounding healthy tissue. Beyond that, the results indicate a clear benefit of the TF over the conventional histogram parameters, especially in patients undergoing ADT. For definite conclusions, large patient cohorts need to be analyzed, ideally in a multicenter study. Validation of our findings by other research groups would be another asset. Nevertheless, the presented results may lay the foundations for development of auto-segmentation tools. In the field of focal therapy, assessing the impact of ADT on imaging parameters is required, as the number of ADT patients has increased significantly over the past years, while no consequences were drawn for the imaging protocols [31]. Only a few studies so far have addressed the issue of MR imaging of ADT patients at all, and the results were not unanimous. In our study, the observed differences in imaging parameters between the patient groups were subliminal and did not reach statistical significance. However, ADC values for the healthy tissue of the CG tended to be 15–20% lower in patients who received ADT (*p* < 0.05), which is in accordance with [18, 19]. Regarding the tissue classification power, in the ADT group, both ADC and T2w TF were superior to the corresponding common histogram parameters. Another interesting observation was that different T2w TF seemed to be of additive value for the two different patient groups. Moreover, our results show that TF are of additive value for the specificity and sensitivity in the ADT group. However, generalization of the presented data is difficult, since the analysis was performed on pre-defined structures. The given criteria thresholds of Table 1 are probably not applicable to a random delineation, as would be necessary for an auto-segmentation approach. These could be on one hand of very small volume and on the other hand may contain a mixture of tumor and healthy tissue. Moreover, it is probably not possible to extrapolate the given criteria thresholds for other patient groups, although normalization of the imaging data was performed in a pre-processing step. Nevertheless, the potential and value of TF in this context was pointed out, which allows envisioning future clinical applications.

Groenendaal et al. aimed to answer the same research question asked in the present study: “Is tumor delineation possible in patients using hormonal treatment?” [17]. They found that the majority of ADT patients can be treated with a focal boost to a suspicious region inside the prostate, but simultaneously suggest using different imaging thresholds depending on the ADT duration. This is in line with the findings of the present study, as delineation of the focal lesion was feasible for ADT patients. However, with respect to the FLAME trial [1, 2] and based on the results above, it can be hypothesized that boost volumes cannot be defined for ADT patients by the same methods. The benefit of using TF in addition was shown over simple ADC thresholding, which alone will not suffice for automated segmentation approaches for ADT patients. For ADT patients, sensitivity and specificity are heavily reduced in comparison to the no-ADT group when only considering histogram-based information. Therefore, including Haralick TF is recommended as these results are superior to the histogram parameters.

With regard to auto-segmentation, further pre-requisites still need to be met. Haralick TF need to become quantitative and reproducible. Brynolfsson et al. investigated the reproducibility and impact of pre-processing on Haralick TF and presented a method to overcome inherent bias caused by selection of different numbers of grey levels [29, 30]. This method was based on transforming the grey-level co-occurrence matrix into a probability distribution. Hereby, they diminished the discretization that causes this bias. The main advantage of using invariant TF is therefore their easy reproducibility and comparability. Furthermore, for new methodologies of focal lesion detection, so-called “local” TF need to be defined and implemented in order to produce TF-maps. Previous developments and preliminary results in this context are highly encouraging [32, 33]. The sum of many such local TF maps could further be used and processed into a tumor probability map by a sound pre-trained algorithm, similar to the methodology of Prior et al. for multiparametric imaging [34]. Our results indicate that for patients without ADT, histogram parameters like T2w_mean and ADC_mean will be of relevance in this context, while for patients undergoing ADT, also TF like ADC_InvariantAutocorrelation will play an important role. Combination with the powerful machine learning and neural network approaches would be an obvious step to take. Validation and implementation in clinical processes is challenging and will require multi-institutional approaches.

Some limitations of the presented study have to be highlighted. First, the study design was retrospective, which might have led to selection bias. This was addressed by applying the inclusion criterion of ADT at some timepoint during the treatment course, which led to a clinical as well as a population size homogenization of the two patient groups. Another limitation is that two different patient groups were compared, meaning it is not possible to model the effect of ADT on the individual patient. Ideally, a study concept would include prospective design, one MRI before, and one MRI after a defined ADT treatment duration [16]. Next, the study population was relatively small. A larger future prospective study will test the clinical applicability of the presented ADC and T2w-derived TF approach in defining boost volumes for prostate cancer patients with neoadjuvant ADT. Due to the small sample size, no prospective validation in independent patients could be performed. However, 10-fold cross-validation was applied for the classification process which is considered appropriate by most authors and was used by other groups before, e. g., [35]. The process of lesion delineation has a major influence on the analysis and should be handled with great care. In our study, the prostate radiology expert amended approximately 25% of the contours based on biopsy cross-validation. Therefore, the added value of biopsy information should be highlighted and could be included in the delineation process in daily clinical routine for patients with ADT. However, the exact shape of the lesion could not be verified, despite biopsy cross-validation. This would require a prostatectomy after the bpMRI scans and a perfect in-vivo and ex-vivo registration [36–39]. Naturally, such a study design for large patient cohorts is challenging. Nevertheless, slight differences between the delineated lesion and “real” lesion affect only a part of our results. Applying a margin could attenuate this issue. It is possible that very small secondary lesions were not detected at all. But as their volume is small, their influence on the analysis will be limited. A boost to these regions may be less relevant, as the main tumor burden is given by the primary lesion. Some other factors of influence might include different DWI parameters and different type, dose, or duration of ADT [17, 40].

## Conclusion

TF of bpMRI showed superior performance in differentiating healthy from tumor tissue compared to conventional histogram parameters in patients treated with ADT. These promising results motivate further research involving larger patient numbers for future applications in radiotherapeutic boost volume delineation for prostate cancer patients.

## Caption Electronic Supplementary Material

Table S1 Details on T2w and ADC scanning protocols of each patient

Table S2 Characteristic clinical parameters of the two patient sub-groups

Figure S1 The decision tree diagrams for both patient groups and both healthy tissue ROIs
