# Supplementary material for: Impact of androgen deprivation therapy on apparent diffusion coefficient and T2w MRI for histogram and texture analysis with respect to focal radiotherapy of prostate cancer
Source: Strahlenther Onkol. 2018 Nov 26;195(5):402–11. doi: 10.1007/s00066-018-1402-3 (PMC6488548; doi:10.1007/s00066-018-1402-3)
Supplement: Supplementary file 1 — Table S1 Details on T2w and ADC scanning protocols of each patient [file 66_2018_1402_MOESM1_ESM.pdf]

Table S1 Details on T2w and ADC scanning protocols of each patient.

Patient Information					Scanner Details			T2w sequence (tse, transversal)						ADC sequence (2D ep spair, transversal)						
**Group**	**ID**	**Age**	**ADT duration [m]**	**Gleason Score**	**Machine**	**Vendor**	**Field Strengh**	**T2w Slice Thickness**	**T2w Slice Spacing**	**T2w Pixel Spacing**	**T2w TR**	**T2w TE**	**T2w NoA**	**b Values**	**ADC Slice Thickness**	**ADC Slice Spacing**	**ADC Pixel Spacing**	**ADC TR**	**ADC TE**	**ADC NoA**
ADT	1	56	7	4+4=8	Biograph_mMR	Siemens	3	3	3.6	0.625	3200	101	3	0/800 /1400	3.6	3.6	1.238	5300	92	6
	2	73	7	4+3=7	Biograph_mMR	Siemens	3	3	3.6	0.521	4000	104	3	0/800 /1400	3.8	3.8	1.238	5300	92	6
	3	74	3	4+3=7	Biograph_mMR	Siemens	3	3	3.6	0.750	4000	101	3	0/850	3.6	3.6	1.458	3800	56	2
	4	77	1	4+5=9	Biograph_mMR	Siemens	3	3	3.6	0.625	4000	101	3	0/800 /1400	3.6	3.6	1.238	5300	92	6
	5	54	118	4+5=9	Espree	Siemens	1.5	3	3.3	0.521	3880	113	2	50/1000 /1400	6	6.6	1.979	2000	95	2
	6	65	1	4+4=8	Ingenia	Philips	3	3.3	3.63	0.341	4000	110	1	0/600 /1200	5	5.5	1.420	4300	80	3
	7	63	104	3+3=6	TrioTim	Siemens	3	3	3.6	0.625	4000	101	3	0/100/400 /800/1600(calc)	3.6	3.6	1.625	3300	60	8
	8	73	70	3+4=7	TrioTim	Siemens	3	3	3.6	0.625	4000	101	3	0/100/400 /800/1600(calc)	3.6	3.6	1.625	3300	60	8
	9	71	33	5+4=9	TrioTim	Siemens	3	3	3.6	0.625	4000	101	3	0/100/400 /800/1600(calc)	3.6	3.6	1.625	3300	60	8
	10	76	4	4+5=9	TrioTim	Siemens	3	3	3.6	0.625	4000	101	3	0/100/400 /800/1600(calc)	3.6	3.6	1.625	3300	60	8
	11	80	3	3+4=7	TrioTim	Siemens	3	3	3.6	0.625	4000	101	3	0/100/400 /800/1600(calc)	3.6	3.6	1.625	3300	60	8
	12	67	2	4+3=7	TrioTim	Siemens	3	3	3.6	0.625	4000	101	3	0/100/400 /800/1600(calc)	3.6	3.6	1.625	3300	60	8
	13	72	2	4+3=7	TrioTim	Siemens	3	3	3.6	0.625	4000	101	3	0/100/400 /800/1600(calc)	3.6	3.6	1.625	3300	60	8
	14	76	1	3+4=7	TrioTim	Siemens	3	3	3.6	0.625	4000	101	3	0/100/800 /1600(calc)	3.6	3.6	1.625	3300	60	8
no-ADT	15	75		4+5=9	Biograph mMR	Siemens	3	3	3.6	0.703	4000	108	3	0/850	3.6	3.6	1.771	6400	56	2
	16	71		4+4=8	Biograph mMR	Siemens	3	3	3.6	0.521	4000	104	3	0/50 /850	3.6	3.6	1.625	5400	93	8
	17	67		4+4=8	Biograph mMR	Siemens	3	3	3.6	0.521	6160	104	3	0/100 /850	3.6	3.6	1.625	7426	93	8
	18	66		3+3=6	Biograph mMR	Siemens	3	3	3.6	0.625	4000	101	3	0/800 /1400	3.6	3.8	1.238	5300	92	6
	19	64		3+4=7	Skyra	Siemens	3	2.5	2.5	0.625	6650	105	3	50/800 /1600	2.5	2.5	1.864	5540	59	2
	20	65		3+3=6	Skyra	Siemens	3	2.5	2.5	0.625	6650	105	3	50/800 /1600	2.5	2.5	1.864	5540	59	2
	21	73		3+4=7	TrioTim	Siemens	3	3	3.6	0.625	4000	101	3	0/100/400 /800/1600(calc)	3.6	3.6	1.625	3300	60	8
	22	75		4+4=8	TrioTim	Siemens	3	3	3.6	0.625	4000	101	3	0/100/400 /800/1600(calc)	3.6	3.6	1.625	3300	60	8
	23	68		3+4=7	TrioTim	Siemens	3	3	3.6	0.625	4000	101	3	0/100/400 /800/1600(calc)	3.6	3.6	1.625	3300	60	8
	24	79		4+5=9	TrioTim	Siemens	3	3	3.6	0.625	4000	101	3	0/100/400 /800/1600(calc)	3.6	3.6	1.625	3300	60	8
	25	79		5+4=9	TrioTim	Siemens	3	3	3.6	0.625	4000	101	3	0/100/400 /800/1600(calc)	3.6	3.6	1.625	3300	60	8
	26	75		4+5=9	TrioTim	Siemens	3	3	3.6	0.625	4000	101	3	0/100/400 /800/1600(calc)	3.6	3.6	1.625	3300	60	8
	27	70		4+3=7	TrioTim	Siemens	3	3	3.6	0.625	4000	101	3	0/100/400 /800/1600(calc)	3.6	3.6	1.625	3300	60	8
	28	74		3+4=7	Verio	Siemens	3	4	4	0.688	4000	111	2	0/100 /800/1400	3.6	3.6	1.742	3300	60	8

tse = turbo spin echo
TR= repetition time
TE= echo time
NoA= number of averages

ep= echoplanar
spair = spectral attenuated inversion recovery
(calc)= extrapolated value (not measured)
