# Supplementary material for: Impact of androgen deprivation therapy on apparent diffusion coefficient and T2w MRI for histogram and texture analysis with respect to focal radiotherapy of prostate cancer
Source: Strahlenther Onkol. 2018 Nov 26;195(5):402–11. doi: 10.1007/s00066-018-1402-3 (PMC6488548; doi:10.1007/s00066-018-1402-3)
Supplement: Supplementary file 2 — Table S2 Characteristic clinical parameters of the two patient sub-groups [file 66_2018_1402_MOESM2_ESM.pdf]

Table S2		no-ADT	ADT
**age [years]**		**70±7**	**71±6**
**PSA [ng/ml]**	**initial**	**23±24 (excl. one outlier>200)**	**21±25 (excl. one outlier>200)**
	**nadir***	**0.02 (0.00; 0.13)**	**0.12 (0.05; 0.30)**
**Gleason Score**	**total**	**7.6±1.0**	**7.6±1.0**
	GS 1	3.6±0.6	3.8±0.6
	GS 2	4.0±0.7	3.8±0.7
**Volume [ccm]**	**lesion**	**1.8 (0.7; 7.9)**	**1.3 (0.4; 4.8)**
	**PZ**	**7 (7; 17)**	**9 (6; 13)**
	**CG**	**23 (18; 40)**	**16 (13; 23)**

Table S2 Characteristic parameters of the two patient sub-groups. With exception of the PSA nadir, none of the parameters differed significantly in between patient subgroups.
Mean±SD is given for normal distributed parameters, median (1st quartile; 3rd quartile) for non-normal distributed ones. * indicates significant difference ($p<0.05$)
