# Supplementary figures and images for: Impact of androgen deprivation therapy on apparent diffusion coefficient and T2w MRI for histogram and texture analysis with respect to focal radiotherapy of prostate cancer
Source: Strahlenther Onkol. 2018 Nov 26;195(5):402–11. doi: 10.1007/s00066-018-1402-3 (PMC6488548; doi:10.1007/s00066-018-1402-3)

Figure S1 The decision tree diagrams for both patient groups and both healthy tissue ROIs.

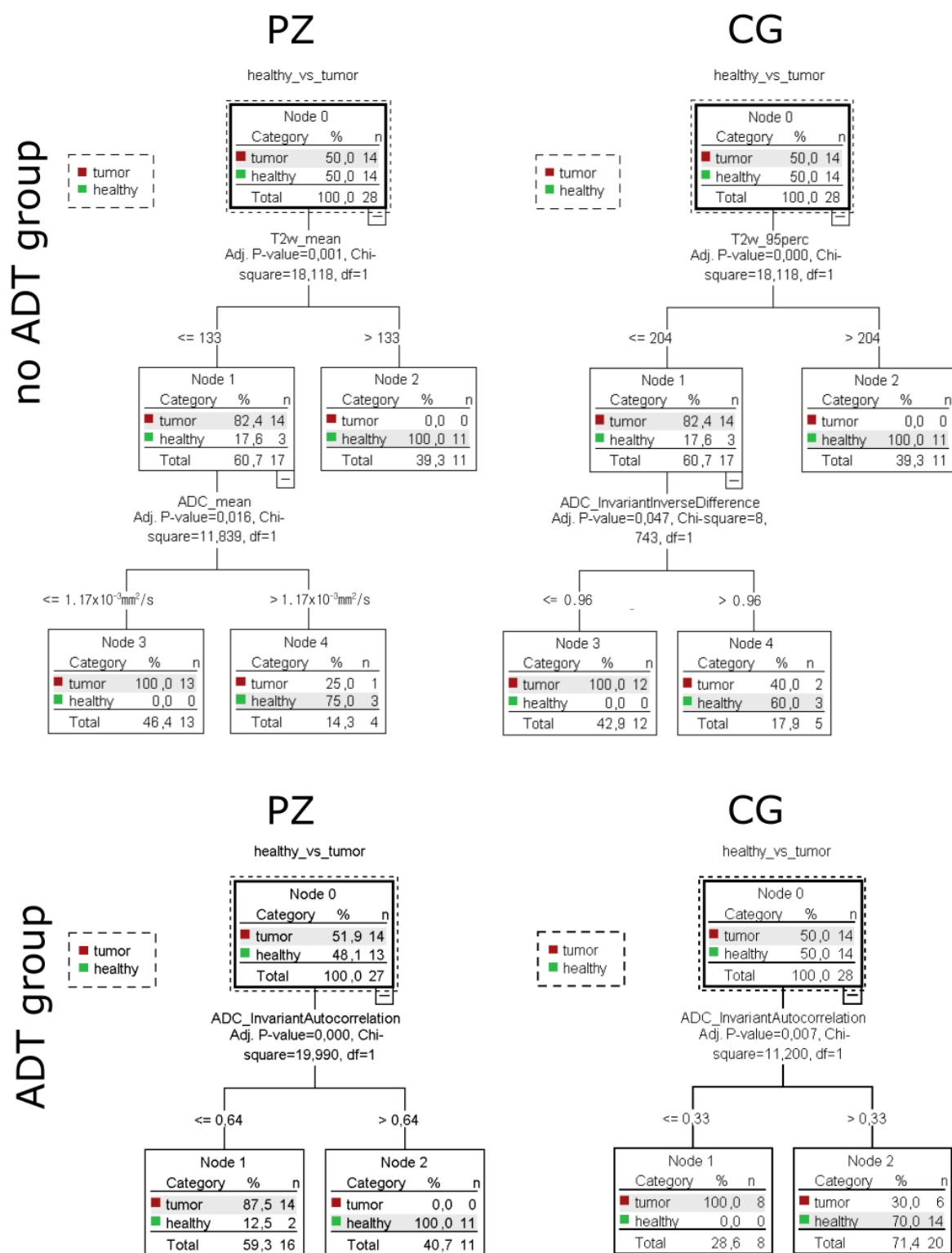

Supplement: Supplementary file 3 — Figure S1 The decision tree diagrams for both patient groups and both healthy tissue ROIs [file 66_2018_1402_MOESM3_ESM.pdf]
